# Combined Pharmacophore Modeling, 3D-QSAR, Homology Modeling and Docking Studies on CYP11B1 Inhibitors

**DOI:** 10.3390/molecules20011014

**Published:** 2015-01-09

**Authors:** Rui Yu, Juan Wang, Rui Wang, Yong Lin, Yong Hu, Yuanqiang Wang, Mao Shu, Zhihua Lin

**Affiliations:** 1 School of Pharmacy and Bioengineering, Chongqing University of Technology, Chongqing 400054, China; E-Mails: 379490507@163.com (R.Y.); wangrx1022@163.com (R.W.); huy@cqut.edu.cn (Y.H.); wangyqnn@cqut.edu.cn (Y.W.); 2 College of Bioengineering, Chongqing University, Chongqing 400044, China; E-Mail: wangjuan7986@126.com; 3 School of Chemistry and Chemical Engineering, Chongqing University of Technology, Chongqing 400054, China; E-Mail: linyong@cqut.edu.cn; 4 School of Chemistry and Chemical Engineering, Chongqing University, Chongqing 400044, China

**Keywords:** CYP11B1 inhibitors, 3D-QSAR, pharmacophore model, Cushing’s syndrome

## Abstract

The mitochondrial cytochrome P450 enzymes inhibitor steroid 11β-hydroxylase (CYP11B1) can decrease the production of cortisol. Therefore, these inhibitors have an effect in the treatment of Cushing’s syndrome. A pharmacophore model generated by Genetic Algorithm with Linear Assignment for Hypermolecular Alignment of Datasets (GALAHAD) was used to align the compounds and perform comparative molecular field analysis (CoMFA) with Q^2^ = 0.658, R^2^ = 0.959. The pharmacophore model contained six hydrophobic regions and one acceptor atom, and electropositive and bulky substituents would be tolerated at the A and B sites, respectively. A three-dimensional quantitative structure-activity relationship (3D-QSAR) study based on the alignment with the atom root mean square (RMS) was applied using comparative molecular field analysis (CoMFA) with Q^2^ = 0.666, R^2^ = 0.978, and comparative molecular similarity indices analysis (CoMSIA) with Q^2^ = 0.721, R^2^ = 0.972. These results proved that all the models have good predictability of the bioactivities of inhibitors. Furthermore, the QSAR models indicated that a hydrogen bond acceptor substituent would be disfavored at the A and B groups, while hydrophobic groups would be favored at the B site. The three-dimensional (3D) model of the CYP11B1 was generated based on the crystal structure of the CYP11B2 (PDB code 4DVQ). In order to probe the ligand-binding modes, Surflex-dock was employed to dock CYP11B1 inhibitory compounds into the active site of the receptor. The docking result showed that the imidazolidine ring of CYP11B1 inhibitors form H bonds with the amino group of residue Arg155 and Arg519, which suggested that an electronegative substituent at these positions could enhance the activities of compounds. All the models generated by GALAHAD QSAR and Docking methods provide guidance about how to design novel and potential drugs for Cushing’s syndrome treatment.

## 1. Introduction

Cortisol is a principal glucocorticoid [1] that is not only used in the treatment of inflammation, allergy, collagen diseases, asthma, adrenocortical deficiency, shock, and some neoplastic conditions, but also exhibits many physiological functions in the regulation of metabolism of life substances, blood pressure and cardiovascular function [2]. Biosynthesis of cortisol take place in the adrenal cortex whose final step (conversion from 11-deoxycortisol) is catalyzed by the mitochondrial cytochrome P450 enzyme steroid 11β-hydroxylase (CYP11B1) [1,3]. (Please reorder references numbers, you jumped 2 and 3 before 4)

The secretion of cortisol is precisely controlled by adrenocorticotropic hormone (ACTH) [2,4,5] within the negative feedback cycle of hypothalamic-pituitary-adrenal axis [1]. However, pathological changes in adrenals and the upstream regulating switches can cause an overproduction of cortisol, which is known as Cushing’s syndrome. Cushing’s syndrome patients mainly show a “moon face”, sanguine temperament appearance, obesity, acne, purple lines, high blood pressure, secondary diabetes and osteoporosis,* etc.* It’s a hormonal disorder caused by prolonged exposure to high levels of circulating glucocorticoids such as cortisol [6,7].

Normally, the surgical removal of adrenal or pituitary tumors is used for the treatment of hypercortisolism [8]. However, as mentioned above, CYP11B1 can promote the synthesis of cortisol. Therefore, inhibition of CYP11B1 as the pharmacological approach to block cortisol biosynthesis represents a treatment for Cushing’s syndrome [9]. Inhibitors of cortisol biosynthesis, such as ketoconazole, etomidate, and metyrapone have been used in the clinic [4], however, all of them show severe side effects due to the fact that they are unselective. Metyrapone is the only drug reported to be a relatively selective CYP11B1 inhibitor.

In recent years, a series of mitochondrial cytochrome P450 (CYP) superfamily receptors [10], such as CYP1A2, CYP17, CYP19, CYP11B2 [7,11,12], were used to analysis the combination of ligand compounds in different molecular docking studies [13]. Few studies published so far have used the pharmacophore modeling or 3D-QSAR approaches for modeling ligand interactions with the CYP11B1 receptor [14]. In addition, a three-dimensional model of CYP11B1 has not been published yet. Therefore, in the present study we aimed to build and validate homology models of CYP11B1 [15] and then run a docking procedure which indicates the active groups and atoms of inhibitory compounds. In order to analyse the molecular shape of CYP11B1 inhibitors [14], a series of compounds with good inhibitory activities synthesized by Hartmann [15] were collected to establish 3D-QSAR models using CoMFA and CoMSIA. The combination of the pharmacophore model [16] GALAHAD and CoMFA methods also helped establish the structure-activity relationship (SAR) of CYP11B1 inhibitors [17].

## 2. Results and Discussion

### 2.1. GALAHAD Modeling Results

**Figure 1:**
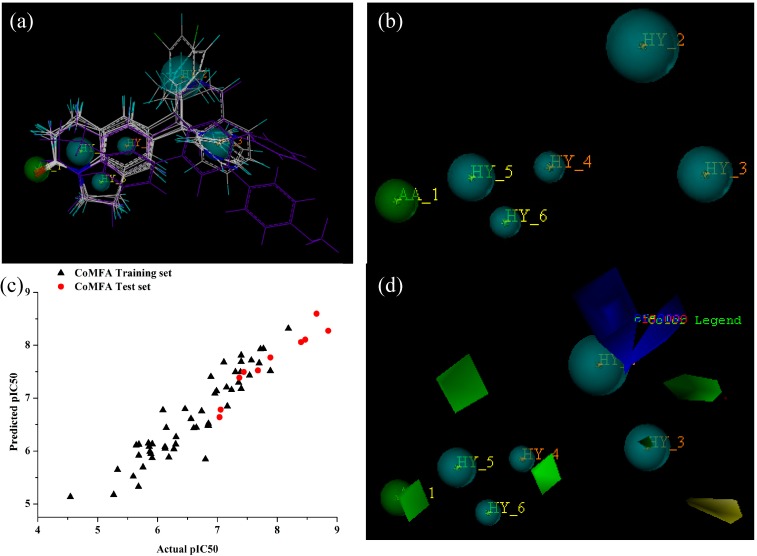
The results generated using GALAHAD modeling method. (**a**) The alignment of 62 CYP11B1 inhibitors; (**b**) The best pharmacophore model generated by the GALAHAD method; (**c**) Observed* versus* predicted pIC_50_ values derived from CoMFA of both training and test sets; (**d**) CoMFA contour maps for the best pharmacophore model.

Once GALAHAD modeling based on the training set compounds was completed (Figure 1a six hydrophobic regions and one acceptor atom (Figure 1b).

They are featured as the cyan balls and the green ball, respectively [18,19]. According to the principals, if all energy parameters had the same level, the Pareto rank would be taken into consideration. Thus, model 1 with a Pareto rank of 0 was selected as the best template to do the CoMFA analysis. The result of this model is Q^2^ = 0.658, R^2^ = 0.959, F = 82.102, SEE = 0.154 and had two components. The model obtained from combined method had an acceptable balance between energy, pharmacophoric coherence and pharmacosteric overlap statistically. Therefore model 1 was used as the template in aligning the full dataset and did partial least square (PLS) analysis [20,21]. The contour plots between observed and predicted activities of all compounds were shown in Table 1. Nearly, all of compounds were located on the trend line (Figure 1c), indicating that the proposed model was able to successfully predict compounds in test set. In the CoMFA study, the contour maps (Figure 1d) of the pharmacophore model indicated that the blue and yellow contours located around site A would be electropositive groups. The yellow contours located at the B site around the hydrophobe group indicated that a bulky substituent would not be tolerated. The green contours around acceptor atoms and hydrophobe groups indicated that a bulky substituent would be tolerated.

**Table 1:** The observed and predicted activities of 62 CYP11B1 inhibitors generated from different modeling methods.

Compounds	pIC_50_			
	Observed	Predicted		
		Pharmacophore	RMS	
		CoMFA	CoMFA	CoMSIA
**1**	6.460	6.795	6.503	6.267
**2**	6.650	6.442	6.724	6.837
**3**	5.910	5.872	6.383	7.355
**4**	6.854	6.476	6.216	6.870
**5**	5.848	6.147	7.039	6.978
**6**	6.128	6.049	6.027	5.972
**7**	5.268	5.174	5.294	5.237
**8**	6.559	6.608	6.635	6.589
**9**	5.595	5.521	5.605	5.488
**10**	6.092	6.773	6.594	6.809
**11**	6.274	6.038	6.286	6.199
**12**	7.056	6.781	7.047	7.100
**13**	6.190	5.883	5.569	5.832
**14**	7.036	6.638	7.112	7.184
**15**	5.874	5.991	5.820	5.838
**16**	6.125	6.082	6.059	6.145
**17**	7.387	7.493	7.384	7.462
**18**	8.398	8.059	8.256	8.199
**19**	7.167	6.846	6.657	6.474
**20**	5.644	6.111	5.710	5.526
**21**	5.914	6.131	6.081	6.216
**22**	7.444	7.493	7.465	7.332
**23**	7.108	7.679	7.499	7.460
**24**	7.770	7.936	8.755	8.459
**25**	8.187	8.317	8.456	8.444
**26**	8.854	8.274	8.730	8.643
**27**	8.658	8.598	8.663	8.569
**28**	8.469	8.105	8.332	8.414
**29**	7.721	7.929	7.230	8.223
**30**	7.398	7.690	7.280	7.521
**31**	7.538	7.430	7.158	7.394
**32**	7.569	7.715	7.188	7.315
**33**	6.959	7.089	7.352	7.539
**34**	7.398	7.810	6.608	7.151
**35**	6.842	6.483	7.030	7.225
**36**	7.155	7.205	7.335	7.278
**37**	7.301	7.494	7.316	7.284
**38**	7.699	7.662	7.777	7.700
**39**	7.886	7.515	7.693	7.596
**40**	6.146	6.438	5.974	6.149
**41**	6.607	6.435	6.367	6.426
**42**	6.801	5.847	6.021	6.082
**43**	6.987	7.134	7.057	6.944
**44**	5.689	6.125	5.698	5.658
**45**	5.890	5.949	5.699	6.199
**46**	6.851	6.518	6.369	7.034
**47**	7.886	7.768	6.820	6.869
**48**	4.544	5.135	5.004	4.603
**49**	5.683	5.329	5.408	5.758
**50**	7.237	7.156	7.095	7.108
**51**	7.367	7.385	7.244	7.113
**52**	6.310	6.264	6.075	6.183
**53**	7.398	7.178	7.525	7.237
**54**	6.738	6.753	6.844	6.712
**55**	6.305	6.128	6.510	6.321
**56**	5.757	5.695	5.681	5.796
**57**	6.893	7.405	7.022	7.331
**58**	5.862	6.085	6.465	7.034
**59**	7.678	7.524	7.821	7.828
**60**	7.357	7.294	6.605	6.842
**61**	5.687	5.917	5.705	5.600
**62**	5.333	5.648	5.336	5.397

### 2.2. 3D-QSAR Modeling Result

**Figure 2:**
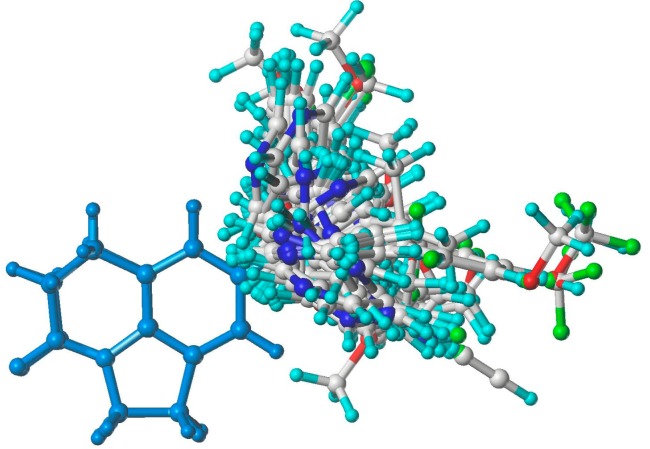
Molecular alignment based on atom root mean square (RMS) was used in the 3D-QSAR studies.

The superimposition of all molecules aligned with a common substructure is shown in Figure 2. The aligned molecules were used to generate CoMFA and CoMSIA models which were developed using a combination of different fields, and the statistically significant models were reported with statistical parameters shown in Table 2.

**Table 2:** Summary of validation statistics for CoMFA and CoMSIA Models generated based on the RMS.

Method	Q^2^	R^2^	N	SEE	F
CoMFA + SE	0.666	0.978	6	0.159	270.441
CoMSIA + S	0.510	0.833	6	0.441	29.904
CoMSIA + E	0.416	0.869	5	0.385	49.217
CoMSIA + H	0.446	0.852	6	0.414	34.641
CoMSIA + D	-	-	-	-	-
CoMSIA + A	0.219	0.729	6	0.562	16.074
CoMSIA + SE	0.531	0.955	6	0.229	126.791
CoMSIA + SH	0.626	0.890	6	0.357	48.766
CoMSIA + SA	0.487	0.830	6	0.445	29.213
CoMSIA + EH	0.446	0.882	6	0.361	70.933
CoMSIA + EA	0.520	0.924	6	0.298	72.500
CoMSIA + HA	0.443	0.873	6	0.384	41.344
CoMSIA + SEH	0.547	0.912	6	0.312	98.306
CoMSIA + SEA	0.699	0.962	6	0.211	150.046
CoMSIA + SHA	0.595	0.916	6	0.312	65.746
CoMSIA + EHA	0.632	0.958	6	0.220	138.387
CoMSIA + SEHA	0.721	0.972	6	0.180	209.908

The CoMFA model using both steric and electrostatic fields gave Q^2^ of 0.666, R^2^ of 0.978, F of 270.441 and SEE of 0.159 values with six components. In the CoMSIA study, the first five models using a single field indicated that the steric field is the most important one. The combination of the steric, electrostatic, hydrophobic, and electrostatic fields led to the S + E + H + A model (Q^2^ = 0.721, N = 6), providing the best overall model. The best model led to the highest R^2^, F and the lowest SEE (R^2^ = 0.972, F = 209.908 and SEE = 0.180). For these reasons, we considered S+E+H+A to be the best possible combination.

### 2.3. Predictive Power of 3D-QSAR Analyses

**Figure 3:**
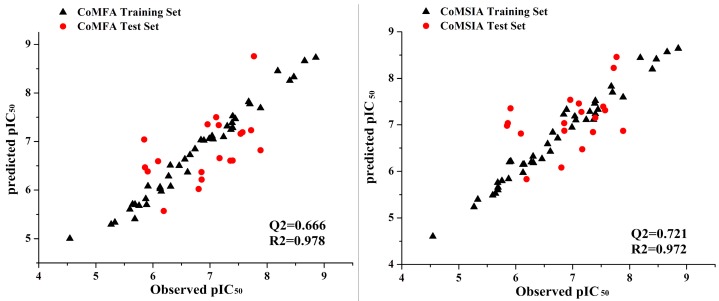
Observed* versus* predicted pIC_50_ values of both training and test sets using CoMFA and CoMSIA in 3D-QSAR mode.

CoMFA and CoMSIA analysis developed QSAR models using the training set of CYP11B1 inhibitors. The predicted and observed activities of these compounds were obtained by using the best model that was given in Table 1. The contour plots between observed and predicted bioactivities of training and test set were shown in Figure 3. Most of compounds were located near the trend line, implying the proposed model is able to successfully predict the activities of compounds, which indicated that these 3D-QSAR models are reliable and powerful in predicting pIC_50_ values.

**Figure 4:**
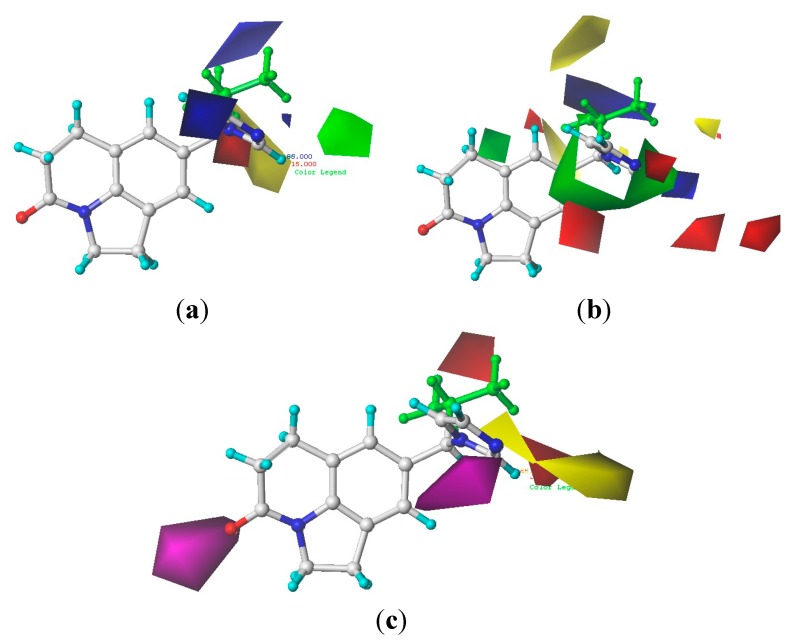
CoMFA and CoMSIA contour maps displayed using the most potent compound **26**. (**a**) CoMFA steric and electrostatic contour map (green indicates favored, yellow indicates disfavored, blue indicate favoreds, red indicates disfavored); (**b**) CoMSIA steric and electrostatic contour map (green indicates favored, yellow indicates disfavored, blue indicates favored, red indicates disfavored); (**c**) CoMSIA hydrophobic and acceptor contour map (yellow indicates favored, white indicates disfavored, magenta indicates favored, red indicates disfavored).

The 3D contour maps were generated to represent the 3D-QSAR results produced by the CoMFA and CoMSIA methods. The different field contributions of COMFA and COMSIA models were illustrated with compound **26** (Figure 4). The results indicated that most of the contours were located at the right side of the compound structures (A and B groups). In the CoMFA model, the contributions of the steric and electrostatic fields to activity were 40.9% and 59.1%. The yellow and blue contours located at the A site indicated that a bulky substituent would not be tolerated and electropositive groups would be favorable [22]. However, bulky substituents and electropositive groups would not be favorable to the B group (Figure 4a) [23]. In the CoMSIA model, the contributions of the steric, electrostatic, hydrophobic and acceptor fields were 13.2%, 40.7%, 22.0% and 24.2%, respectively. The percentages of different fields indicated that the steric and electrostatic fields almost make the same contribution. The combination of these four fields provided the most predictive model for CYP11B1 inhibitors, and the electrostatic field was the most significant for bioactivity prediction. The contour maps of the steric and electrostatic fields of CoMSIA (Figure 4b) were generally in accordance with the field distribution of the CoMFA maps (Figure 4a). The hydrophobic and hydrogen bond acceptor field contour maps of CoMSIA implied that hydrogen bond acceptor substituents were disfavored at the A and B sites, while, hydrophobic groups were favored at the B site (Figure 4c) [24]. The small and electropositive substituent such as an aliphatic amine group would be tolerated at the A site. Small, electronegative and hydrophobic substituent such as trifluoromethanesulfonamide group would be tolerated at B site.

### 2.4. Homology Modeling Result

**Figure 5:**
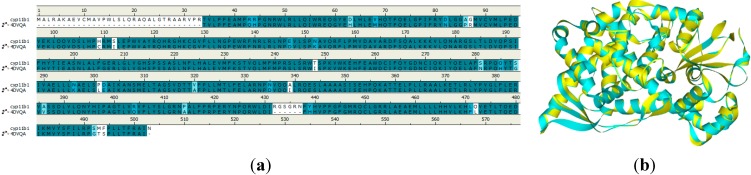

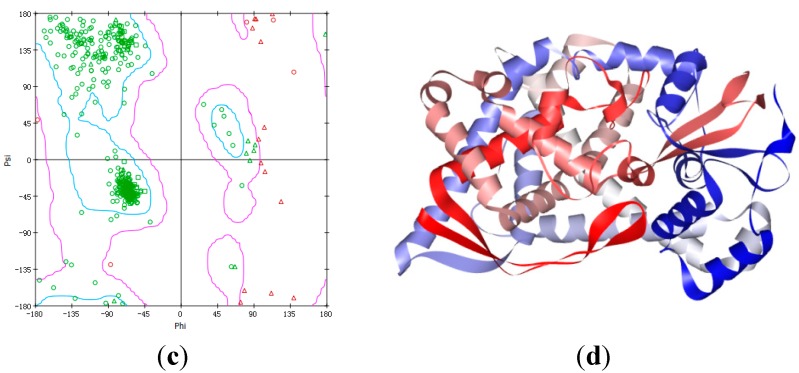
Structural-based alignment of CYP11B1 and the template PDB ID: 4DVQ-A. (**a**) The sequence shaded in cyan represents sequence similarity; (**b**) Superposition of the predicted CYP11B1model onto to the template PDB ID: 4DVQ-A, yellow indicates template, blue indicates predicted model; (**c**) Ramachandran Plot of the best CYP11B1 model; (**d**) Structure of the best predicted model.

The length of the CYP11B1 sequence was 574 aa and the most suitable template was the A chain of the CYP11B2 protein (PDB code: 4DVQ). The BLASTP alignment between the CYP11B2 template and CYP11B1 sequences is shown in Figure 5a, revealing 81% identity and 95% consensus similarity. Then, after the structural-based alignment of CYP11B1 from 4DVQ-A, the initial 3D structure of CYP11B1 was obtained from a homology modeling procedure shown in Figure 5d. Evaluation of the homology models consists of Profiles_3D scores and Ramachandran plot analysis [25] (Figure 5c).

Profiles_3D scores shown in the Ramachandran Plot of the CYP11B1 model showed a good distribution of 574 amino acid residues of CYP11B1. About 97.0% of the residues featured with green plots were most favored and an additionally allowed region, and only 3.0% residues featured with red plots were an erroneously allowed region.

### 2.5. Docking Analysis

**Figure 6:**
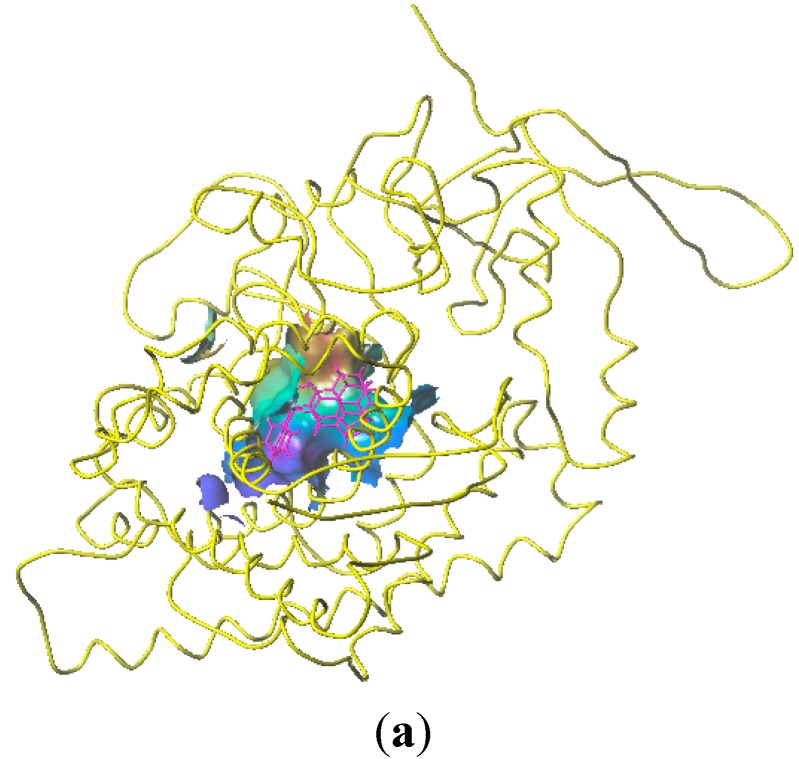

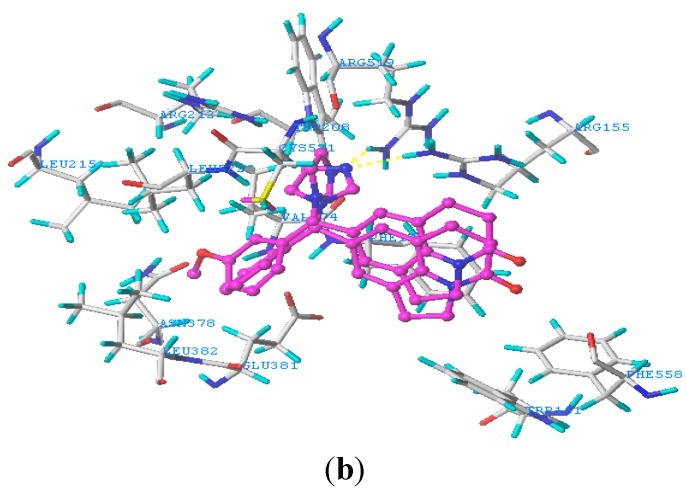
The Surflex-dock result. (**a**) The docking complex of compounds **33**, **37** with CYP11B1; (**b**) Binding mode of compounds **33**, **37** with CYP11B1.

Sixty two (62) CYP11B1 inhibitors were evaluated by docking scores after Surflex-dock. According to the rule, compounds with scores above 7 were considered as active in the docking study. In this paper compounds **33** and **37** scored 7.8544 and 7.1932 (Figure 6a,b) and showed a similar docking mode in the active site of the receptor. The binding cavity of CYP11B1 is formed by residues Arg155, Phe175, Arg519, Cys521, Leu522. Hydrogen atoms of the amino group of residue Arg155 and Arg519 are found near N3 of the imidazolidine ring, thus suggesting that electronegative substituents at these positions could enhance the activities of compounds binding to CYP11B1 [26,27].

These results matched well with the electrostatic contour maps of the CoMFA and CoMSIA models suggesting that an electronegative substituent such as a trifluoromethanesulfonamide would be positive at the B site as mentioned above (Figure 1d and Figure 4a,b).

## 3. Experimental Section

### 3.1. Data Set

The structures of 62 CYP11B1 inhibitors and their biological activities were taken from Hartmann *et al*. [1,12,18]. The inhibitory activity IC_50_ values (nM) were converted to the reciprocal logarithmic values (pIC_50_ = −log IC_50_) which range from 5.26 to 8.52. All the calculations and analyses of CYP11B1 inhibitors were performed using Discovery Studio 3.0 (Accelrys Software Inc., San Diego, CA, USA, 2011) and SYBYL-X2.1 software (Tripos Inc., St. Louis, MO, USA, 2014) [17,28].

### 3.2. GALAHAD

Pharmacophore modeling by GALAHAD [11,29] alignment and CoMFA analysis serve as useful tools to produce pharmacophore models of CYP11B1 inhibitors and predict the inhibitory properties of compounds [30,31]. Ten structurally representative compounds with high activities marked with “#” were selected as training set (Table 3) to generate pharmacophore models using the GALAHAD program. The default parameters were used for the GALAHAD runs with the population size turned to 75 and max generations turned to 30. The generated models were evaluated by the test set of CoMFA modeling based on the pharmacophore alignment [30,32]. Therefore the most reasonable pharmacophore model was selected to predict the bioactivities of components and analyse the structures of CYP11B1 inhibitors [33].

**Table 3:** The structures and bioactivity values of active compounds.

Compound	Skeleton	R	IC_50_ (nM)	pIC_50_
**1**	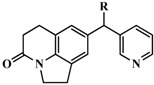	OH	347	6.4597
**2**		H	224	6.6498
**3** *		=CH_2_	1230	5.9101
**4** *		Me	140	6.8539
**5** *		*i*-Pr	1420	5.8477
**6**		Ph	745	6.1278
**7**		Ph,OH	5399	5.2677
**8**		2-MeOPh	276	6.5591
**9**		3-MeOPh	2539	5.5953
**10** *		4-MeOPh	810	6.0915
**11**		3-FPh	532	6.2741
**12** ^#^		4-FPh	88	7.0555
**13** *		3-ClPh	646	6.1898
**14** ^#^		4-ClPh	92	7.0362
**15**		3-CH_3_Ph	1336	5.8742
**16**	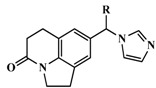	OH	750	6.1249
**17**		=CH_2_	41	7.3872
**18** ^#^		Me	4	8.3979
**19** *		Ph	68	7.1675
**20**		2-MeOPh	2270	5.6440
**21** *		3-MePh	1220	5.9136
**22** ^#^		3-ClPh	36	7.4437
**23** *		4-FPh	78	7.1079
**24** *	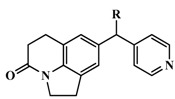	Me	17	7.7696
**25**		Et	6.5	8.1871
**26** ^#^		*i*-propyl	1.4	8.8539
**27** ^#^		*c*-propyl	2.2	8.6576
**28** ^#^		*c*-butyl	3.4	8.4685
**29** *		=CH_2_	19	7.7212
**30** *		2-FPh	40	7.3979
**31** *		3-FPh	29	7.5376
**32** *		4-FPh	27	7.5686
**33** *		3-MeOPh	110	6.9586
**34** *		4-MeOPh	40	7.3979
**35**		3-CNPh	144	6.8416
**36** *		4-CNPh	70	7.1549
**37**		Ph	50	7.3010
**38**		2-furanyl	20	7.6990
**39**		2-thienyl	13	7.8861
**40**	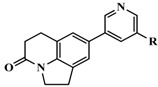	H	715	6.1457
**41**		OMe	247	6.6073
**42** *		OEt	158	6.8013
**43**		OiPr	103	6.9872
**44**		OH	2045	5.6893
**45**		F	1288	5.8901
**46**		CF_3_	141	6.8508
**47** ^#^*	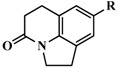	4-isoquinoline	13	7.8861
**48**		5-pyrimidine	28546	4.5445
**49**		1-imidazole	2077	5.6826
**50**	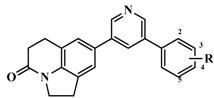	H	58	7.2366
**51** ^#^		2-F	43	7.3665
**52**		3-F	490	6.3098
**53**		4-F	40	7.3979
**54**		2,5-F	183	6.7375
**55**		3,4-F	496	6.3045
**56**		3,5-F	1748	5.7575
**57**		2-OMe	128	6.8928
**58** *		3-OMe	1374	5.8620
**59** ^#^		4-OMe	21	7.6778
**60** *		3-OH	44	7.3565
**61**		3-OCF_3_	2058	5.6866
**62**		3-CF_3_	4646	5.3329

Molecules marked with *: belong to the test set of 3D-QSAR method; Molecules marked with ^#^: belong to the test set of GALAHAD method.

### 3.3. 3D-QSAR Modeling

**Figure 7:**
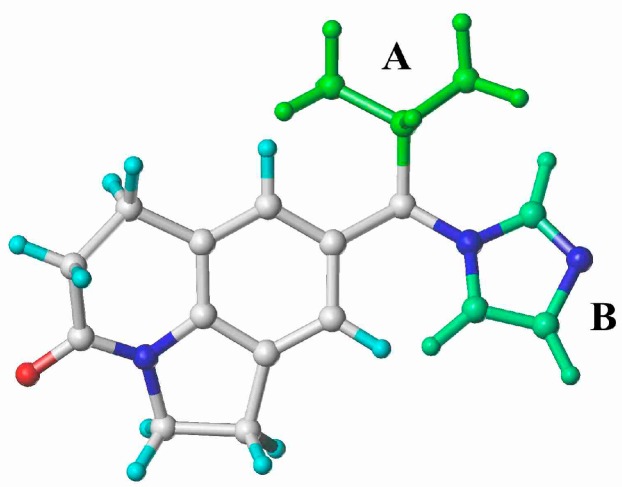
The compound with the highest activity of CYP11B1 inhibition (compound **26**) combined with A and B groups.

The structures of CYP11B1 inhibitors used for the 3D-QSAR study were randomly divided into a training set (42 molecules) and a test set (20 molecules) [34] (Table 1). All structures were energy minimized using the Powell gradient algorithm under the Tripos force field with Gasteiger-Marsili atomic partial charges. The most potent CYP11B1 inhibitor (compound **26**) was selected as the alignment template (Figure 7), which was also used as template to build all the other inhibitors with the atom root mean square (RMS) approach.

3D-QSAR models were constructed by using CoMFA and CoMSIA methods based on the molecular alignment. The default values of the parameters of the CoMFA and CoMSIA methods were used. The CoMFA method was performed using steric and electrostatic fields with standard 30 kcal/mol cutoffs. In the CoMSIA study, besides steric and electrostatic fields, three other different fields were calculated: hydrophobic, hydrogen bond donor, and hydrogen bond acceptor [34,35,36]. A series of models were constructed with an increasing number of partial least squares (PLS) analysis factors. The numbers of components in the PLS models were optimized by using the cross-validated correlation coefficient (Q^2^), non-cross-validated correlation coefficient (R^2^), standard error estimate (SEE) and F-statistic values (F*)*,* etc*., which were obtained from the leave-one-out (LOO) cross-validation procedures [37,38]. According these parameters, the best model was chosen to predict bioactivities of compounds. In this work the best 3D-QSAR model was graphically represented by field contour maps, and the coefficients were generated using the StDev⁄Coeff field type.

### 3.4. Homology Modeling and Docking Analysis

The amino acid sequence of CYP11B1 (GenBank: AAH96285.1) was obtained from the National Center for Biotechnology Information Database (NCBI) [39]. Identification of candidate templates was performed by sequence similarity search using BLAST search (NCBI Sever) protocol with default values and each target was searched and downloaded from the NCBI database. The CYP11B1 sequence was aligned to the templates and homology models of CYP11B1 built with the default parameters. Subsequently, the models were analyzed based on Profiles_3D scores and Ramachandran plots which indicate the percentage of amino acids located in the disallowed regions. The interaction of small molecule ligands with a protein, which implied atomic-detail accuracy including position and conformation, was obtained from the docking procedure. Docking and scoring were performed using the Sybyl-X2.1 software (Tripos Inc., St. Louis, MO, USA, 2014) Surflex-Dock method. The docking receptor CYP11B1 was constructed as described in Homology modeling, while the ligands set consisted of all 62 inhibitors. Polar hydrogen atoms were added to both protein and ligand structures. The other parameters of Surflex-Dock methods were used as default values. The best docking pose was selected according to the total score.

## 4. Conclusions

CYP11B1 plays a crucial role in the biosynthesis of cortisol which can cause a series of diseases known as Cushing’s syndrome. Therefore, CYP11B1 inhibitors that can be regarded as a pharmacological approach to block cortisol biosynthesis have become another treatment for Cushing’s syndrome. GALAHAD is a novel pharmacophore screening module, which generates models to analyse the pharmacophore features of ligands. A CoMFA study based on the pharmacophore (GALAHAD) alignment has been developed to derive the structure-activity relationships, which also indicate the interaction between CoMFA fields and pharmacophore features of ligands.

In this study, CoMFA and CoMSIA models were built using the alignment based on the atom root mean square (RMS), which explored the structure-activity relationship of the CYP11B1 inhibitors. These models with excellent consistency manifested good predictive ability for the test compounds. The contour maps also identified the important features contributing to interactions between the different fiedls and the active site of CYP11B1 inhibitors.

Homology modeling method was used to construct a 3D model of the CYP11B1 protein. Then, the Surflex-Dock analysis was used to evaluate the binding activities between the protein and ligand compounds. The binding site indicated the interaction and combination between ligand groups and amino acid residues, which acted as a guide in the prediction and design of CYP11B1 inhibitors.

The combined pharmacophore modeling and 3D-QSAR modeling methods indicated that small and electropositive substituents would be tolerated at the A site. Meanwhile, small, electronegative and hydrophobic substituents would be better at the B site. The docking results indicated that electronegative substituents at the B position could enhance the activities of compounds binding to CYP11B1.
